# Viscoelastic properties of bovine articular cartilage attached to subchondral bone at high frequencies

**DOI:** 10.1186/1471-2474-10-61

**Published:** 2009-06-04

**Authors:** Geoffrey R Fulcher, David WL Hukins, Duncan ET Shepherd

**Affiliations:** 1 School of Mechanical Engineering, University of Birmingham, Edgbaston, Birmingham, B15 2TT, UK

## Abstract

**Background:**

Articular cartilage is a viscoelastic material, but its exact behaviour under the full range of physiological loading frequencies is unknown. The objective of this study was to measure the viscoelastic properties of bovine articular cartilage at loading frequencies of up to 92 Hz.

**Methods:**

Intact tibial plateau cartilage, attached to subchondral bone, was investigated by dynamic mechanical analysis (DMA). A sinusoidally varying compressive force of between 16 N and 36 N, at frequencies from 1 Hz to 92 Hz, was applied to the cartilage surface by a flat indenter. The storage modulus, loss modulus and phase angle (between the applied force and the deformation induced) were determined.

**Results:**

The storage modulus, *E'*, increased with increasing frequency, but at higher frequencies it tended towards a constant value. Its dependence on frequency, *f*, could be represented by, *E' *= *Alog*_*e *_(*f*) + *B *where *A *= 2.5 ± 0.6 MPa and *B *= 50.1 ± 12.5 MPa (mean ± standard error). The values of the loss modulus (4.8 ± 1.0 MPa mean ± standard deviation) were much less than the values of storage modulus and showed no dependence on frequency. The phase angle was found to be non-zero for all frequencies tested (4.9 ± 0.6°).

**Conclusion:**

Articular cartilage is viscoelastic throughout the full range of frequencies investigated. The behaviour has implications for mechanical damage to articular cartilage and the onset of osteoarthritis. Storage modulus increases with frequency, until the plateau region is reached, and has a higher value than loss modulus. Furthermore, loss modulus does not increase with loading frequency. This means that more energy is stored by the tissue than is dissipated and that this effect is greater at higher frequencies. The main mechanism for this excess energy to be dissipated is by the formation of cracks.

## Background

**Figure 1:**
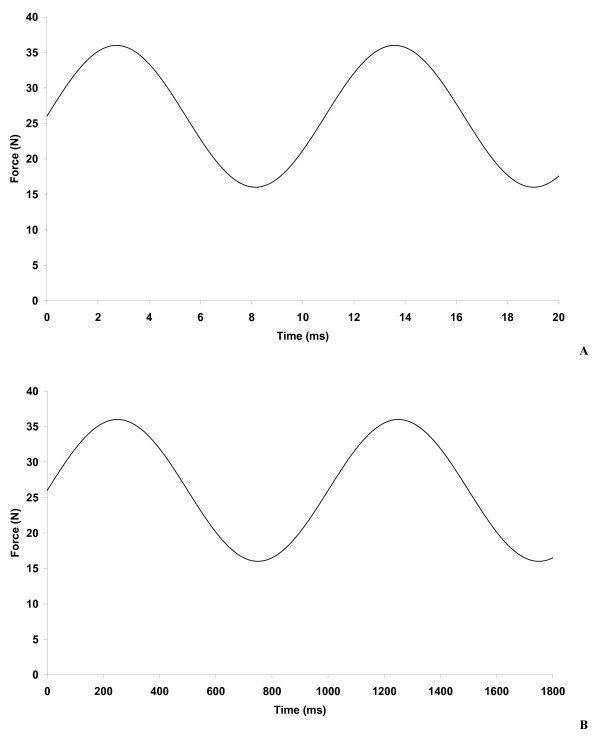
**Sinusoidally varying force A) at 92 Hz (rise time 5.4 ms); B) at 1 Hz (rise time 500 ms)**.

This paper presents the viscoelastic properties of bovine articular cartilage, attached to subchondral bone, determined at loading frequencies of up to 92 Hz. Articular cartilage is a viscoelastic material [1,2], but its exact behaviour under the full range of physiological loading frequencies is unknown. The rise time of the heel strike force for most of the population is typically 100–150 ms [3]. However, Radin *et al*. [4] have identified a subset of the population that have a rise time of the heel strike force to be of sufficiently short duration to create impulsive loading. These rapid heel strike force rise times can be in the range 5–25 ms [3,5]. It has been suggested that these high rise times at heel strike could be implicated in the onset of osteoarthritis [6]. The effect of rapid heel strike force can be investigated by determining the viscoelastic properties of articular cartilage at high frequencies, as shown in Figure 1. The rise time of the force is given by the time from the trough to the peak of the sine wave, that is:

(1)

where *f *is the frequency of the sine wave. Thus a sinusoidally varying force with a frequency of 92 Hz would have a rise time 5.4 ms, with a rise time of 500 ms for a frequency of 1 Hz. This matches the rise time seen *in vivo*, but the loading time is of shorter duration which is typically 1000 ms in a human joint during walking. Until recently, materials testing machines have not had the capability of operating at high enough frequencies to be able to investigate heel strike rise times of short duration.

When a sinusoidally varying compressive stress, is applied to a viscoelastic material the resulting strain will be out of phase by an angle *δ*. A viscoelastic material is characterised by a storage modulus, *E'*, and a loss modulus, *E''*. The storage modulus represents the elastic part of the response (where energy is stored and used for elastic recoil of the specimen when a stress is removed) and the loss modulus represents the viscous response (where energy is dissipated and the material flows). The magnitude of the complex modulus is given by [7,8]:

(2)

while the phase angle is given by:

(3)

A phase angle of 0° would indicate that a material is purely elastic, while a purely viscous material would have a phase angle of 90°.

## Methods

Bovine knee joints, from animals less than 30 months old, were obtained from Fresh Tissue Supplies (East Sussex, UK). On arrival in the laboratory they were wrapped in tissue paper soaked in Ringer's solution, sealed in a plastic bag and stored in a freezer at -40°C until they were required for testing. Freezing has been previously shown not to change the mechanical properties of articular cartilage [9,10]. Once a joint was defrosted, it was dissected and a specimen of articular cartilage-on-bone, was cut using a saw. A specimen was either a medial or lateral tibial plateau, with the total thickness (cartilage and bone) typically 20 mm, to allow sufficient bone for secure fixation in the test rig. All cartilage was visibly normal to the naked eye. The specimen was then secured into a custom-made test rig using acrylic cement (WHW Plastics, Hull, UK).

The viscoelastic response of the articular cartilage was determined using a Bose ElectroForce 3200 testing machine, operated under the control of WinTest dynamic mechanical analysis (DMA) software (Bose Corporation, ElectroForce Systems Group, Minnesota, USA). Full details on the testing machine have been previously given [11] and the Bose testing machines have been used to test a wide range of materials [8,11-14], including articular cartilage [15].

An indenter of diameter 5.2 mm (but with a small radius at the edge to prevent damage to specimens) was attached to the actuator of the testing machine. The test rig, containing the specimen, was attached to the base of the testing machine. The custom-made test rig enabled small adjustments to be made to the orientation of the specimen so that the region of articular surface being tested was perpendicular to the direction of compression by the indenter. The cartilage specimens were left to equilibrate for around 10 minutes before testing and maintained in Ringer's solution throughout the tests, which were undertaken at room temperature (21–23°C), as in previous studies [16,17].

A sinusoidally varying compressive force of between 16 N and 36 N (giving a maximum nominal stress of 1.7 MPa) was applied during the testing. The resulting displacement was also sinusoidally varying with the amplitude of the displacement in the range 0.012 to 0.093 mm. At the beginning of each test specimens were subjected to 1500 cycles of loading at 25 Hz and 3000 cycles at 50 Hz (with a rest period of 60 s between frequencies) to allow the specimens to stabilise before data collection. These values were determined during preliminary experiments, but are similar to the conditions of 1200 cycles previously used to reach a steady state [15]. During the testing, where data were collected for measuring the viscoelastic properties of the cartilage, the sinusoidally varying compressive force was applied for 2 s at each frequency tested in the range 1–92 Hz. The frequency of 1 Hz simulated a heel strike force rise time of 500 ms, while 92 Hz simulated a rise time of 5.4 ms. At the end of each frequency tested, the applied force returned to the mean value of 26 N and was maintained for a dwell time of 2 s before the testing at the next frequency was undertaken. The testing involved starting at 1 Hz and increasing the frequency; preliminary tests showed that increasing the frequency from 1 Hz to 92 Hz gave the same results as decreasing the frequency from 92 Hz to 1 Hz, with the settings used. The specimens tested, and the frequencies to which they were subjected, are shown in Table 1. Six to nine sites on the central region of four tibial plateaux were tested; it was not the intension of the current study to investigate the full topographical variation in viscoelastic properties over the whole cartilage surface.

**Table 1:** Details of cartilage sites tested and summary of the results for storage modulus, loss modulus and phase angle

**Point**	**Thickness (mm)**	**Frequency range (Hz)**	**Storage modulus curve fit**				**Loss modulus (MPa)**	**Phase angle (°)**
			***A *(SE)**	***B *(SE)**	***R*^2^**	***p***	**Mean (SD)**	**Mean (SD)**
1	2.9	1 to 92	3.4 (0.2)	69.3 (0.5)	0.99	0	6.6 (0.6)	4.8 (0.2)
2	2.1	1 to 92	3.0 (0.2)	66.4 (0.7)	0.97	0	6.7 (0.3)	5.1 (0.5)
3	1.1	1 to 92	2.8 (0.3)	81.2 (1.0)	0.93	0	5.3 (0.5)	3.4 (0.5)
4	2.8	1 to 80	1.7 (0.2)	47.6 (0.8)	0.93	0.002	4.5 (0.8)	4.9 (0.7)
5	2.5	1 to 80	2.3 (0.3)	56.1 (0.8)	0.95	0.001	5.7 (0.5)	5.2 (0.4)
6	1.7	1 to 80	2.5 (0.2)	57.3 (0.7)	0.97	0	5.7 (0.3)	5.1 (0.6)
7	1.1	1 to 72	1.9 (0.2)	75.4 (0.5)	0.97	0	5.0 (0.7)	3.6 (0.7)
8	1.9	1 to 72	2.8 (0.2)	62.3 (0.5)	0.99	0	6.4 (0.4)	5.3 (0.7)
9	2.4	1 to 72	2.8 (0.2)	59.8 (0.6)	0.98	0	5.9 (0.3)	5.0 (0.3)
10	1.8	1 to 72	2.3 (0.04)	45.9 (0.1)	1.00	0	4.9 (0.3)	5.4 (0.8)
11	1.7	1 to 72	2.1 (0.1)	56.3 (0.3)	0.99	0	4.8 (0.3)	4.4 (0.2)
12	1.7	1 to 72	1.8 (0.06)	39.0 (0.2)	0.99	0	3.6 (0.2)	4.6 (0.2)
13	2.4	1 to 72	2.1 (0.1)	43.0 (0.3)	0.98	0	4.2 (0.3)	4.9 (0.2)
14	2.7	1 to 72	1.8 (0.1)	38.5 (0.4)	0.98	0	3.9 (0.2)	5.1 (0.2)
15	2.4	1 to 72	1.9 (0.07)	42.8 (0.2)	0.99	0	4.1 (0.2)	4.9 (0.2)
16	2.5	1 to 72	1.5 (0.1)	36.1 (0.3)	0.98	0	3.4 (0.4)	4.8 (0.3)
17	2.7	1 to 60	3.3 (0.2)	55.8 (0.6)	0.98	0	5.5 (0.3)	4.9 (0.3)
18	2.8	1 to 60	3.2 (0.2)	53.5 (0.5)	0.99	0	5.9 (0.4)	5.3 (0.5)
19	3.2	1 to 50	1.9 (0.2)	37.9 (0.4)	0.98	0	4.2 (0.3)	5.7 (0.3)
20	1.7	1 to 30	3.1 (0.2)	46.0 (0.5)	0.98	0	5.3 (0.3)	5.7 (0.1)
21	2.4	1 to 30	3.3 (0.3)	55.2 (0.7)	0.97	0	5.2 (0.3)	4.7 (0.2)
22	2.6	1 to 30	2.7 (0.2)	49.1 (0.5)	0.97	0	4.8 (0.2)	4.9 (0.2)
23	2.7	1 to 30	2.2 (0.2)	36.4 (0.4)	0.97	0	3.2 (0.2)	4.4 (0.1)
24	1.7	1 to 18	3.0 (0.2)	45.2 (0.4)	0.98	0	5.0 (0.2)	5.6 (0.6)
25	2.6	1 to 18	2.3 (0.1)	35.1 (0.2)	0.98	0	3.6 (0.2)	5.3 (0.1)
26	3.1	1 to 18	2.7 (0.2)	37.8 (0.4)	0.97	0	3.3 (0.2)	4.4 (0.1)
27	2.9	1 to 18	3.0 (0.2)	46.7 (0.4)	0.97	0	4.0 (0.2)	4.4 (0.1)
28	2.8	1 to 12	3.1 (0.2)	43.9 (0.3)	0.99	0	4.7 (0.1)	5.5 (0.3)
29	1.9	1 to 10	2.4 (0.3)	32.3 (0.6)	0.92	0.001	3.6 (0.1)	5.6 (0.3)

The storage modulus is described by a line of the form *E' *= *A*log_*e *_(*f*) + *B*, where *f *represents frequency. SE is the standard error of the coefficients *A *and *B*. *R*^2 ^is a squared correlation coefficient and shows how well the line fits the data points. If *p *< 0.05 it indicates that the line is statistically significant. Loss modulus and phase angle results given as mean and standard deviation (SD).

During all tests the applied force and the displacement were measured. After a test was completed the WinTest software calculated the dynamic stiffness, k*, (the ratio of the force to displacement; units N/mm) and phase angle, *δ*, (the phase difference between the force and displacement). The magnitude of the moduli (*E'*, *E''*) were calculated by dividing the stiffness by a shape factor, given by:

(4)

where *d *is the diameter of the indenter (5.2 mm) and *t *is the articular cartilage thickness. The thickness of the articular cartilage was determined using an established technique in which a sharp needle was pushed through the cartilage layer and into the underlying bone, using a testing machine [3,18]. The storage modulus (*E'*) and the loss modulus (*E''*) were calculated from:

(5)

(6)

## Results

**Figure 2:**
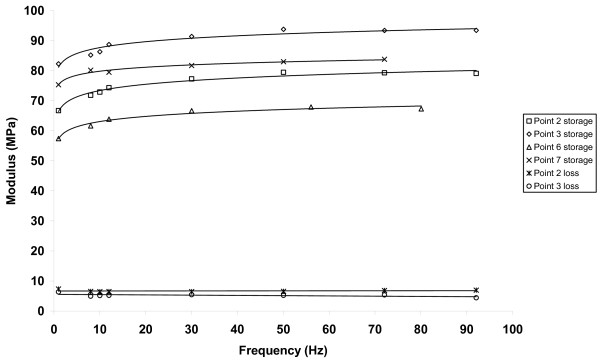
**Storage and loss moduli against frequency, for frequencies of up to 92 Hz**.

**Figure 3:**
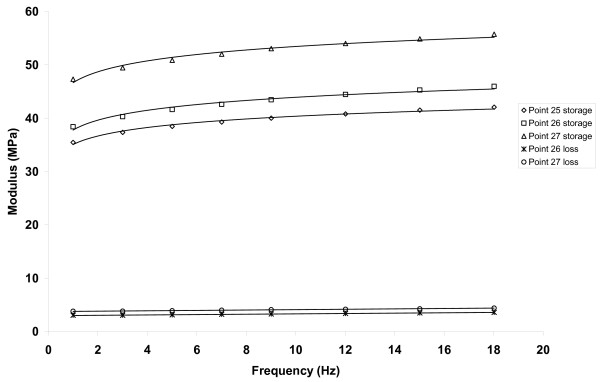
**Storage and loss moduli against frequency, for frequencies of up to 18 Hz**.

The storage modulus of articular cartilage, *E'*, increased with increasing frequency, *f*. Figure 2 shows the storage modulus determined at frequencies of up to 92 Hz. For point 3 it can be seen that *E' *increased from 82 MPa at 1 Hz to 93 MPa at 92 Hz. It can been seen that at higher frequencies *E' *reached a plateau. A line of the form *E' *= *A*log_*e *_(*f*) + *B *was found to fit the data well for all sites tested; values of *A *and *B *were determined to obtain the best fit to the experimental results for each site (Table 1). From this line it can be seen that increase *E' *with increasing *f *is greater a lower *f *values, since its slope , is inversely proportional to *f*. Figure 3 shows the storage modulus against frequency plotted at frequencies of up to 18 Hz. For all sites tested the constants *A *and *B *were in the ranges 1.5 to 3.4 MPa (mean = 2.5 MPa, standard deviation = 0.6 MPa) and 32.3 to 81.2 MPa (mean = 50.1 MPa, standard deviation = 12.5 MPa), respectively. No correlation was found between articular cartilage thickness and the values of the constants *A *and *B*.

The loss modulus of articular cartilage was much less than the storage modulus over all frequencies tested (Figures 2 and 3) and showed no dependence on frequency. Indeed, the loss modulus had a fairly constant value across the frequencies for all tests. The mean value of loss modulus for each site tested was in the range 3.2 to 6.7 MPa (for all sites, mean = 4.8 MPa, standard deviation = 1.0 MPa). The phase angle was found to be non-zero for all frequencies tested with the mean values for each site tested being in the range 3.4 to 5.7° (for all sites, mean = 4.9°, standard deviation = 0.6°). Table 1 summarises the results of the storage modulus, loss modulus and phase angle for all the tested samples of articular cartilage-on-bone.

## Discussion

The results show that the storage modulus of articular cartilage increases with increasing frequency, but that this increase levels out into a plateau before the highest frequency of 92 Hz was reached. This is the first time that this observation has been made because the viscoelastic properties of articular cartilage have not previously been measured at such high frequencies. An increase in storage modulus with increasing frequency has been seen in previous studies, but only up to a maximum testing frequency of 8 Hz [19] or 10 Hz [20,21]. In the current study it was observed that although for each individual site tested, the higher the frequency the higher the storage modulus, the rate of change of modulus is highest at low frequencies. At higher frequencies the storage modulus tended towards a constant value. This result is consistent with the observation that E*, defined in equation 2, for articular cartilage was almost insensitive to increasing rates of loading at high strain rates (strain rates of 0.1 to 1000 s^-1^) [22]. Previous studies have also shown that the loss modulus of articular cartilage was much less than the storage modulus and that it was insensitive to loading frequency [20,21]; however, in these studies the frequency did not exceed 10 Hz.

The dependence of storage modulus on frequency, in which storage modulus increases but then levels out to a plateau, is characteristic of a material undergoing a glass transition [23]. In a glass transition, a soft material (low storage modulus) changes to a glassy material (high storage modulus) with increasing temperature. However, in biological systems, it is more relevant to consider the material at a constant temperature and for the transition to be driven by increasing loading frequency [24]. Similar behaviour has been observed when testing synthetic hydrogels in shear [24] and in compression [8]. It is then not surprising that the same behaviour occurs in articular cartilage since its hydrated proteogyclan component is effectively a natural hydrogel [25]. Articular cartilage is a composite material in which collagen fibrils provide tensile reinforcement; in the different zones of the cartilage, the orientations of the fibrils can be identified with the directions in which the tissue needs to withstand tensile stress [26]. It is, therefore, difficult to explain its properties solely in terms of the properties of the proteoglycan gel. One possible reason for increased stiffness, in a cyclic test, is that the time taken for appreciate fluid flow, in the proteoglycan gel, is short compared with the periodic time associated with high-frequency sinusoidal loading. However, fluid flow is not the only possible mechanism for viscous effects of this kind. The beginning of a similar transition has been observed in the silicone elastomers used for small joint replacement implants [13].

The differing behaviour of storage modulus and loss modulus with increasing frequency, has implications for mechanical damage to articular cartilage and the onset of osteoarthritis. Storage modulus increases with frequency, until the plateau region is reached, and has a higher value than loss modulus. Furthermore, loss modulus does not increase with loading frequency. This means that more energy is stored by the tissue than is dissipated and that this effect is greater at higher frequencies. The main mechanism for this excess energy to be dissipated is by the formation of cracks. This expectation is consistent with the formation of cracks *in vitro*, when articular cartilage has been subjected to impact loading where the energy is high [15,27], and *in vivo *using animal models [28]. The investigation of crack growth under high frequency loading will be the subject of a separate study.

This study shows that the range of values for storage modulus was variable for the different specimens tested and therefore it was not appropriate to quote values as a mean and standard deviation. Many previous studies have observed differences in the modulus of articular cartilage between different specimens and indeed topographical variations in the modulus have been observed over the surface of the same joint in human ankle, hip and knee joints [16,29,30].

For all tests there was a mean phase angle between stress and strain in the range 3.4 to 5.7° indicating that articular cartilage is viscoelastic throughout the full range of frequencies tested (1 to 92 Hz). A previous study by Tanaka *et al*. [21] showed cartilage-on-bone samples to be viscoelastic at a frequency of 10 Hz of loading. Some previous studies have suggested that articular cartilage is elastic (i.e. the phase angle is zero) when it has been loaded at 40 Hz [17] and it has even been suggested that articular cartilage is elastic at loading of 1 Hz [31]. One reason for the differences between these two studies and the current study is that in the current study the cartilage remained on the bone during testing; in the other two studies the cartilage was separated from the bone. Another reason could be limitations in the test apparatus used in the previous studies. The current study used a Bose ElectroForce 3200 testing machine (Bose Corporation, ElectroForce Systems Group, Minnesota, USA) which uses a linear motor, that incorporates a moving magnet design that enables high frequency dynamic testing. The machine has previously been used in measuring the viscoelastic properties of hydrogels, up to a frequency of 20 Hz [8] and elastomers, up to a loading frequency of 100 Hz [13].

## Conclusion

Articular cartilage is viscoelastic throughout the full range of frequencies investigated (1 to 92 Hz). The behaviour has implications for mechanical damage to articular cartilage and the onset of osteoarthritis. Storage modulus increases with frequency, until the plateau region is reached, and has a higher value than loss modulus. Furthermore, loss modulus does not increase with loading frequency. This means that more energy is stored by the tissue than is dissipated and that this effect is greater at higher frequencies. The main mechanism for this excess energy to be dissipated is by the formation of cracks.

## Competing interests

The authors declare that they have no competing interests.

## Authors' contributions

GRF carried out the experimental work, data analysis and revising the manuscript. DWLH participated in the study design, data analysis and revising the manuscript. DETS devised the study, participated in data analysis and drafted the manuscript. All authors read and approved the final manuscript.

## Pre-publication history

The pre-publication history for this paper can be accessed here:


